# BLAINDER—A Blender AI Add-On for Generation of Semantically Labeled Depth-Sensing Data

**DOI:** 10.3390/s21062144

**Published:** 2021-03-18

**Authors:** Stefan Reitmann, Lorenzo Neumann, Bernhard Jung

**Affiliations:** 1 Virtual Reality and Multimedia Group, Institute of Computer Science, Freiberg University of Mining and Technology, 09599 Freiberg, Germany; jung@informatik.tu-freiberg.de; 2 Operating Systems and Communication Technologies Group, Institute of Computer Science, Freiberg University of Mining and Technology, 09599 Freiberg, Germany; lorenzo.neumann@informatik.tu-freiberg.de

**Keywords:** machine learning, depth-sensing, LiDAR, Sonar, virtual sensors, labeling, Blender

## Abstract

Common Machine-Learning (ML) approaches for scene classification require a large amount of training data. However, for classification of depth sensor data, in contrast to image data, relatively few databases are publicly available and manual generation of semantically labeled 3D point clouds is an even more time-consuming task. To simplify the training data generation process for a wide range of domains, we have developed the *BLAINDER* add-on package for the open-source 3D modeling software Blender, which enables a largely automated generation of semantically annotated point-cloud data in virtual 3D environments. In this paper, we focus on classical depth-sensing techniques Light Detection and Ranging (LiDAR) and Sound Navigation and Ranging (Sonar). Within the *BLAINDER* add-on, different depth sensors can be loaded from presets, customized sensors can be implemented and different environmental conditions (e.g., influence of rain, dust) can be simulated. The semantically labeled data can be exported to various 2D and 3D formats and are thus optimized for different ML applications and visualizations. In addition, semantically labeled images can be exported using the rendering functionalities of Blender.

## 1. Introduction

**Figure 1:**
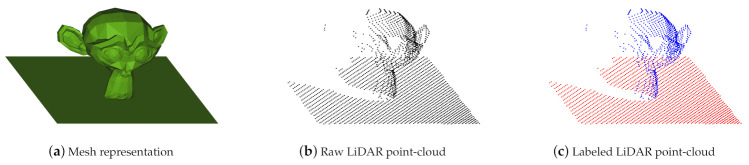
In (**a**) the mesh representation of a certain object (*Suzanne*) is shown. (**b**) comprises the virtually measured point-cloud, while in (**c**) the point-cloud is labeled for classification and segmentation tasks.Main steps of synthetic point clouds

Depth sensors have become ubiquitous in many application areas, e.g., robotics, driver assistance systems, geo modeling, and 3D scanning using smartphones. The output of such depth sensors is often used to build a 3D point-cloud representation of the environment. Artificial Intelligence (AI) approaches, often based on ML techniques, then can be used to understand the structure of the environment by providing a semantic segmentation of the 3D point-cloud, i.e., the detection and classification of the various objects in the scene. To train such classifiers, however, large amounts of training data are required that provide labeled examples of correct classifications. In this paper, we propose an approach (https://github.com/ln-12/blainder-range-scanner (accessed on 17 March 2021)) where virtual worlds with virtual depth sensors are used to generate labeled point clouds (Figure 1c) based on 3D meshes (Figure 1a). In addition, we can automatically annotate rendered images for image classification tasks within this pipeline (see Section 4).

Depth-sensing is achieved by means of waves or rays that are sent out by a transmitter, reflected at surfaces and detected again by a receiver. The time difference between emitting and receiving gives information about the distance covered. Various physical principles are used, such as electromagnetic waves (Radar), acoustic waves (Sonar) or laser beams (LiDAR).

For real-world applications, multiple temporally and spatially offset measurements are used to obtain a point-cloud representation of the environment. Three questions are important with respect to such data. These are the *classification* of individual objects, the recognition of the components of objects (*part segmentation*), and the *semantic segmentation* of several objects in a scene.

Procedures of supervised learning require a large amount of training data. Although several such datasets for 3D point clouds exist, their scope is more limited than in the case of 2D camera images which hampers the transfer to custom domains. Thus, the necessary labeling of training data (*labeling*) must be done manually for specific applications.

To avoid the time-consuming manual labeling process of 3D point clouds and thus to provide a tool for rapid generation of ML training data across many domains, we have developed the *BLAINDER* add-on, a programmatic AI extension of the open-source software Blender.

### 1.1. Related Work

One option for automated processing of point clouds with ML, e.g., for segmentation, is unsupervised learning. For example, a cluster analysis can be used to segment a point-cloud into certain parts. Such a procedure is described in [1,2]. The classification of data points into groups (also: *clusters*) is done by grouping elements that are as similar to possible to each other. More detailed information on this can be found in [3].

Supervised learning is usually used for classification tasks and, in contrast to unsupervised learning, requires semantically labeled examples for training. For two-dimensional image data, the learning of classifies from examples is described e.g., in [4,5,6]. Learning of classifiers for the case of three-dimensional data has been investigated e.g., in [7,8,9]. Regardless of the number of dimensions, methods of supervised learning are often used to automatically recognize patterns and relationships. Such an approach, however, requires a large amount of training data with the correct classification for each pixel or point in a scene (pixel-wise or point-wise segmentation). Alternatively, smallest circumscribing rectangles or bounding boxes may be used depending on the needed amount of precision.

Interest in virtual sensors has grown rapidly in recent years. LiDAR simulation in particular has generated scientific interest in recent years. Especially [10,11,12,13,14,15,16,17] should be mentioned here, which have investigated laser depth detection in different contexts, but without labeling the achieved data. Using ray tracing for autonomous car training, ref. [18] produced a sensor simulator based on LiDAR. A similar method was developed by [19], in which synthetic point clouds with semantic labels of detected objects were generated with a ray tracing LiDAR simulator. Ref. [20] proposed a novel LiDAR simulator that augments real point clouds with synthetic obstacles (moving objects, such as vehicles and pedestrians), in contrast to previous simulators that rely on and game engines and CG. Ref. [21] trained a deep neural network using unbounded synthetic data with automatically generated labels. A consideration of weather effects on LiDAR can be found in [22], where a LiDAR simulator with laser beam propagation and energy attenuation in clear weather, fog, rain, and other harsh conditions is proposed. For improvements in robot navigation [23] shows a LiDAR simulator integrated in the development of navigation tasks. Simulations of LiDAR are now highly relevant even in exotic use cases, as read in [24,25].

Virtual sensor technology needs to be simulated in virtual worlds. Virtual environments can not only be implemented in Blender, but also in game engines, such as Unreal Engine 4 [26]. A further simulator for LiDAR measurements is presented in [27]. It was developed based on the game engine Unity. For the simulation of different sensors, commercial solutions exist such as those from the manufacturers *dSPACE* [28] or *Presagis* [29]. Simulators for robots and their environment are ideal for simulating three-dimensional data. Examples are the tools *MORSE* [30], *Gazebo* [31], and *Webots* [32]. The tool *LiDARsim* [33] offers numerous possibilities for the simulation of LiDAR sensors.

We chose Blender as base for *BLAINDER*, as it is completely open-source, provides powerful modeling tools to easily modify the environment, and comes with a plug-in (or add-on) mechanism for extending built-in functionalities with Python code. Comparable applications in Blender are *Blensor* [34] (LiDAR simulation without labeling for an outdated version of Blender) and *BlenderProc* [35] (only image labeling). The Heidelberg LiDAR Operations Simulator (abbr. *Helios*) [36,37] is a free and open-source tool written in JAVA/C++ and enables the generation of classified point clouds. As this is the most similar tool to ours and it is supported by an add-on for the integration of Blender scenes in *Helios* [38], we use it for direct comparison considering accuracy and performance (see Section 5.2.4).

To provide an appropriate range of variations for synthetic data, virtual environments with different content must be created. For this purpose, different repositories exist, which contain a multitude of categorized objects, which can then be integrated into the scene in different ways. There are various training data sets for this purpose, such as *PASCAL VOC* [39], *PASCAL3D+* [40] or *ShapeNet* [41]. A detailed comparison of further synthetic data sets is summarized in [42].

Despite the surge of interest in the field of virtual sensors in recent years, our approach offers novel features. The main added values of our paper to the research field are:

- The sensor system is directly linked to the Blender software and thus to a wide range of options and tools for creating and animating virtual worlds.
- In addition to LiDAR, we have implemented Sonar simulation for the first time with this level of accuracy and consider the specific properties of water bodies and Sonar characteristics.
- Besides labeling the point clouds for LiDAR and Sonar, our tool can generate image annotations and use Blender’s rendering qualities for labeled image data. The export scope of the synthetic data exceeds all publications mentioned.

### 1.2. Structure of This Article

In Section 2, we summarize the physical laws governing depth-sensing. The main focus here is on the differences between optical and sound measurements and the associated physical characteristics. These lead to an appropriate integration of error functions. In Section 3, the implemented modules of the add-on are introduced in detail and their application is demonstrated exemplarily. Elementary in this section are the descriptions of the labeling process, animations and data export. Section 4 contains exemplary applications to demonstrate the functionality. Essential here is the validation, which refers on the one hand to the performance (among other things computation times, output sizes), on the other hand to the accuracy of the simulated sensor technology. Section 6 gives a final outlook and summarizes the results of the work.

## 2. Fundamentals of Depth-Sensing

This chapter details the fundamental concepts of light and sound propagation and reflection under ideal and perturbed conditions as well as the error models used in the implementation of the various depth sensors in BLAINDER.

### 2.1. Light Detection and Ranging (LiDAR)

LiDAR is a common method for optical distance measurement. Generally, passive and active sensor systems can be distinguished. In this article, we focus on active distance measurement, where radiation is introduced into the environment by the measuring device. An example are cameras that can determine distances by means of Time-of-Flight (ToF) or the active triangulation [43].

The following general equation [44] is used to model an optical distance measurement:

(1) $$P_{r}\left(R\right)=E_{p}\frac{c\eta A}{2R^{2}}\cdot \beta \cdot T\left(R\right)$$

In Equation (1) $P_{r}\left(R\right)$ refers to the power measured by the sensor in watts at a distance of *R*. $E_{p}$ is the energy emitted by the transmitter in joules, *c* stands for the speed of light in $\frac{m}{s}$, η indicates the efficiency of the system, *A* stands for the size of the aperture, β is the backscatter coefficient of the target object and T(R) indicates the signal reduction by the transmission medium. If the reflection at the target object follows Lambert’s law, β applies:

(2) $$\beta =\frac{\gamma }{\pi }$$

In this equation 0<γ<1, which is the target’s surface reflectance. Furthermore, the signal reduction on the transmission path can be calculated by:

(3) $$T\left(R\right)=\exp\left(-2\int_{0}^{R}\alpha \left(r\right)dr\right)$$

Here α(r) is the extinction coefficient of the transmission medium. This coefficient is influenced by particles, such as rain in the air, for example [44]. In the case of rain, this coefficient can be assumed to be evenly distributed so that the integral becomes the constant αz. Furthermore, the values for the emitted energy, the speed of light, the efficiency as well as the aperture are constant and can be summarized as

(4) $$C_{s}=\frac{cE_{p}A\eta }{2}$$

Since $C_{s}$ is also constant, $P_{n}=P_{r}/C_{s}$ applies to the relative measured energy. Thus, Equation (1) is simplified to [45]:

(5) $$P_{n}\left(R\right)=\frac{\beta }{R^{2}}\;e^{-2\alpha z}$$

### 2.2. Sound Navigation and Ranging (Sonar)

Sonar is a distance-measurement method based on sound waves. Like LiDAR, measurements devices can be divided into active and passive (In passive Sonar, the target object itself rather than the sensing device emits a sound signal. This signal can be identified by its characteristic signal profile) Sonar. Again, only the more common active type is considered in BLAINDER. Here, the transmitter emits a signal in the form of a sound wave. The sound wave is reflected at the target object and registered at the receiver. The time difference between signal transmission and reception provides information about the distance ($d=\frac{v\times \Delta t}{2}$ with propagation speed *v*). Active sonars are used, for example, to locate schools of fish in waters or to map the bottom of waters [46].

The following equation applies to active sonars [46] regarding energy conservation:

(6) SL−2TL+TS−NL+DI+PG>RT

The values are given in the unit decibel (abbreviation dB). This is used for sound pressure, among other things, and is defined as:

(7) $$I_{dB}=10\;log_{10}\left(I\right)$$

where *I* stands for the linear intensity and $I_{dB}$ for the intensity on the logarithmic scale in dB. In Equation (6) SL is the source level of the signal at the transmitter. This is reduced by transmission loss TL and noise level NL. The reflection strength of the target TS (target strength) is added to the signal. This indicates how much of the incident sound energy is reflected by the target object [47]. The directivity index DI (also known as array gain) and the gain due to signal processing PG (processing gain) are also added. The directivity index indicates how well the receiver can filter out signals from a specific direction with respect to noise [48]. An echo signal is always detected if the resulting signal is above the reception threshold of the RT receiver [49]. Further explanations can be found in [50].

#### 2.2.1. Spreading of Waterborne Sound

For the simulation of Sonar measurements, the characteristic properties of water bodies must be considered. Decisive for the propagation of a sound wave in water is the velocity of propagation. This is not homogeneous within a body of water. Instead, the velocity *c* [m/s] varies depending on the temperature *T* [°C], the salinity *S* [‰] and the depth *D* [m] [51]. An empirically determined formula for the calculation of *c* is [46,52]:

(8) $$c=1449.2+4.6T-0.055T^{2}+0.00029T^{3}+\left(1.34-0.010T\right)\left(S-35\right)+0.016D$$

### 2.3. Interaction of Light and Sound with Matter

When light or sound waves hit an object, they may be reflected, refracted or absorbed. Both light and sound travel through space as waves. When interacting with matter, however, light behaves like a collection of many individual particles. This phenomenon is also known as wave-particle dualism. Because of the similarity in propagation, many physical laws apply to both types of waves. Generally, approximations are used to model the interaction with a reduced computational effort. The propagation of a wave is linear if the medium is homogeneous. Therefore, the ray model is used in the following for simplification.

#### 2.3.1. Reflection

For the reflection of a ray the law of reflection in Equation (9) applies. In the ideal reflection (also: specular reflection) the incident light beam is reflected exclusively in the direction of $\theta_{2}$.

(9) $$\mathrm{angle}\;\mathrm{of}\;\mathrm{incidence}\;\theta_{1}=\mathrm{angle}\;\mathrm{of}\;\mathrm{reflection}\;\theta_{2}$$

The prerequisite for this is a reflective, smooth surface, such as a mirror. Starting from a normal perpendicular to the surface on which the beam is reflected, the angle of incidence $\theta_{1}$ between the vector of the incident light beam and the normal is equal to the angle of reflection $\theta_{2}$ between the vector of the reflected light beam and the normal [53].

If, on the other hand, a material is rough, such as blotting paper, we speak of diffuse reflection. In Lambert’s illumination model, the incident light is scattered evenly in all directions.

In reality, reflections are much more complex than can be described by these two models. Depending on the material, horizontal angle (also: azimuth angle) and vertical angle (also: polar angle) of the incident and reflected light beam as well as the wavelength of the light play a role. With the help of the bidirectional reflectance distribution function (BRDF) *R* these factors can be taken into account. For the intensity of a reflected light beam $I_{r}$ Equation (10) applies. $I_{e}$ stands for the intensity of the incident light beam.

(10) $$I_{r}=R\cdot I_{e}\cdot cos\left(\theta_{1}\right)$$

Analogous to the distribution function *R* to describe the reflection, a distribution function to describe the transmission bidirectional transmittance distribution function (BTDF) can be set up [53]. Both functions are combined in Blender as the bidirectional scattering distribution function (BSDF) [54].

#### 2.3.2. Refraction

Refraction is a phenomenon that occurs when a wave passes between two media with different refractive indices. The relationship between the angles of incidence ($\theta_{1}$, $\theta_{2}$) and the refractive indices ($n_{1}$, $n_{2}$) can be described by *Snell’s law* of refraction [55]:

(11) $$\frac{\sin\theta_{1}}{\sin\theta_{2}}=\frac{n_{2}}{n_{1}}$$

It should be noted that part of the light is reflected during refraction depending on the angle of incidence. The larger $\theta_{1}$ is, the more light is reflected. This phenomenon is known as the *Fresnel effect*. Transparent surfaces, such as glass panes, behave like mirrors at large angles of incidence. To simulate the Fresnel effect, Equation (12) can be used to determine the reflectivity *R*. The transmittance *T*, i.e., the part of the light that is allowed to pass through the fabric, is given by T=1−R [56].

(12) $$R=\frac{1}{2}\left(\frac{sin^{2}\left(\theta_{3}-\theta_{1}\right)}{sin^{2}\left(\theta_{3}+\theta_{1}\right)}+\frac{tan^{2}\left(\theta_{3}-\theta_{1}\right)}{tan^{2}\left(\theta_{3}+\theta_{1}\right)}\right)$$

This also applies to sound waves. However, the Fresnel effect cannot be applied equivalently. Instead, the transmittance α is determined as follows [52]:

(13) $$\alpha =\frac{4\rho_{1}\cdot c_{1}\cdot \rho_{2}\cdot c_{2}\cdot cos\;\theta_{1}\cdot cos\;\theta_{3}}{{\left(\rho_{2}\cdot c_{2}\cdot cos\;\theta_{1}+\rho_{1}\cdot c_{1}\cdot cos\;\theta_{3}\right)}^{2}}$$

$\rho_{1}$ and $\rho_{2}$ stand for the density of the respective medium and $c_{1}$ and $c_{2}$ for the corresponding propagation speeds. The angle of refraction $\theta_{3}$ can be determined by means of Equation (11) and the angle of incidence $\theta_{1}$ and the refractive indices *n*.

#### 2.3.3. Measurement Errors Induced by Reflection and Refraction

**Figure 2:**
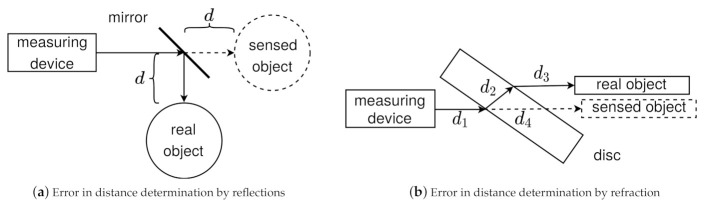
(**a**) The sketch shows a measuring device, a mirror, and a target object. A beam is emitted from the measuring device in the direction of the mirror. There it is deflected by 90° and hits an object. At this object the beam is reflected and returns on the same path. (**b**) The drawing depicts a measuring device, a glass plane and a target object. The beam hits the glass pane, is refracted at the air-glass and glass-air boundaries and then runs parallel to the original direction.

Figure 2a illustrates a measurement error when using reflective materials. You can see a measuring device, a mirror and a target object in top view. The measuring device emits a beam in the direction of the mirror where it is deflected by 90°. Then the beam hits an object at a distance *d* from the mirror. The surface of the object reflects the beam back to the measuring device via the mirror. The measuring device has only knowledge about the direction of the emitted beam and the time difference until the re-arrival of the beam. Reflections or refractions cannot be measured. The measuring point is therefore assumed to be in the direction of the emitted beam with the distance calculated by equation $d=\frac{v\times \Delta t}{2}$ (propagation speed *v* depends on transmission medium). In the case of reflection from a mirror described above, the target appears to be at the position of the virtual object.

A second measurement error is shown in Figure 2b. You can see a measuring device, a glass pane, a target object and an emitted beam. Since glass has a higher refractive index than air, the beam is refracted towards the surface normal (also: perpendicular) and continues in a straight line within the glass pane. At the exit, a second refraction occurs and the beam is refracted away from the surface normal. Since the refractive indices at entrance and exit are the same, but in reverse, the beam runs parallel to the original direction behind the glass pane. Again, the measuring instrument cannot detect the refraction. The determined distance from the measuring device to the target object $d_{4}$ is composed of the distance between the measuring device and the glass pane $d_{1}$ and the length of both refracted beams $d_{2}$ and $d_{3}$. The position thus determined is illustrated by the virtual object.

#### 2.3.4. Atmospheric Influences (Dust, Rain, Fog, Snow) on LiDAR

Optical distance measurement is subject to various external factors that influence the measurement result. Various systems have already been developed to reduce this influence [57]. Nevertheless, in the following some rules for modeling these factors will be presented to be able to simulate systems without such technology. In Equation (3) the reduction of the signal due to propagation in the transmission medium is described. Particles in the air (dust particles, rain, fog, snow) can cause such a reduction of the signal strength or a random error for the measured distance. To model such errors, the influence of rain on a LiDAR measurement was investigated in [45,58]. Therefore, a potential relation between the extinction coefficient α and the rainfall rate $R_{f}$ is formulated:

(14) $$\alpha =a{\left(R_{f}\right)}^{b}$$

In different investigations the coefficients *a* and *b* were empirically determined. Although a=0.01 and b=0.6 are assumed in [45], the values a=0.063 and b=0.37 are determined for the experiments in [58]. A generally valid statement for each kind of precipitation is not given in both publications. In the current BLAINDER implementation the values suggested by [45] are used. An adjustment of these parameters is however possible, if deemed appropriate for an application.

Moreover, influencing the intensity, rain also affects the measured distance *R*. The error is approximated using a Gaussian distribution (see also Section 2.3.5) [45]:

(15) $$R^{\prime }=R+N\left(0,0.02R{\left(1-e^{-R_{f}}\right)}^{2}\right)$$

In addition to precipitation particles in the form of raindrops, dust particles in the air can also reduce the intensity of the light beam and contaminate the sensor. The more particles there are between the measuring device and the target object or on the optics of the measuring device, the more the signal is reduced on its way. The effect of dust on the sensor optics is investigated experimentally for example in [59]. A correlation between the number and type of dust particles in the air and the influence on the measurement is presented in [60]. For simplification, it is assumed that all particles are equally large and homogeneously distributed within the dust cloud. When the light beam is propagated within the dust cloud, scattering leads to a reduction in intensity.

To determine the reduction of the signal during propagation, Equation (3) is extended to

(16) $$T\left(R\right)=\exp\left(-2\;\pi \;r^{2}\;n\;L_{d}\right)$$

Here *r* stands for the radius of a particle in meters, *n* for the number of particles per cubic meter and $L_{d}$ for the length of the dust cloud between the measuring device and the target object in meters. The backscatter of the dust cloud is determined using Equation (17).

(17) $$\beta =\frac{r^{2}\;n}{4}$$

The parameters are equivalent to those in Equation (16) [60]. Other weather phenomena to be considered include fog and snow. In [61] the authors investigate the influence of precipitation on LiDAR measurements and formulate corresponding calculation models. However, the results of the publication could not be reproduced in the context of this work and are therefore subject of future considerations.

Another influencing factor is the ambient temperature. In [62] the influence of this on the measured distance is determined experimentally. The relationship between the two quantities is linear. Within the framework of the exemplary test arrangement, a temperature change of 80 Kelvin led to a measurement error of 1.02 m. A model for the simulation was not formulated. Therefore, such influences are considered in the form of systematic perturbations (see Section 2.3.5).

#### 2.3.5. Random Measurement Error

During the use of sensors for distance measurement a lot of errors can occur. Measurement errors due to reflection and refraction have already been discussed in the previous Section 2.3.3. Furthermore, every measurement of physical quantities is subject to a random error. This error is caused, for example, by tolerances of the measuring device or external influences such as temperature, air pressure or wind. In the case of the distance-measurement methods considered here, the errors in the measuring devices and the influence of particles in the air play a major role. Random measuring errors cannot be predicted. However, occurring perturbations can be modeled statistically using a probability density function f(x). It can be applied if the measured values are distributed symmetrically around a maximum and the probability of occurrence decreases with increasing distance. Often the Gaussian normal distribution is used:

(18) $$f\left(x\right)=\frac{1}{\sqrt{2\pi }\sigma }e^{-\frac{1}{2}{\left(\frac{x-\mu }{\sigma }\right)}^{2}}$$

The characteristic values of the function are the standard deviation σ and variance $\sigma^{2}$ and the expected value μ. The form of the function is similar to the typical shape of a bell. For this reason, it is also referred to as Gaussian bell curve [63].

## 3. Modules of the Implementation

The *BLAINDER* add-on for the open-source software Blender implements the depth-sensing principles presented in Section 2. Virtual LiDAR and Sonar sensors in combination with the powerful 3D modeling and animation capabilities allow the generation of semantically labeled training data for ML algorithms. In contrast to the Blensor project [34], the source code of Blender is not modified by our add-on and re-compilation of Blender is not necessary. Thus, the *BLAINDER* add-on can be used without re-configuration after updates of the Blender software.

**Figure 3:**
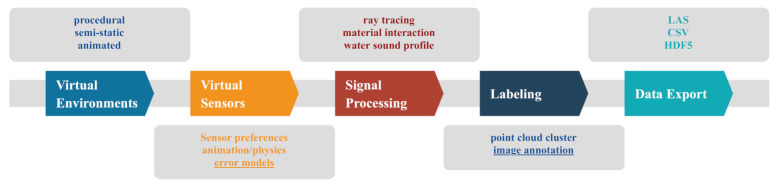
The graph includes the modularized structure of the BLAINDER add-on. The individual submodules are presented in detail in the following chapters. The modularization allows complete adaptability of the add-on to other specific problems.

Figure 3 depicts the main modules of our add-on. BLAINDER provides functionalities for modeling and animation of virtual environments (Section 3.1), virtual sensors (Section 3.2), signal processing (Section 3.3), labeling (Section 3.4) and data export (Section 3.5). Two modules are colored in the diagram. The underlined module of virtual sensors—error models—is subject to ongoing work and the current implementation is considered preliminary. The underlined module of labeling—image annotation—provides a useful additional feature that is however beyond the scope of the present article.

### 3.1. Scene Construction/Virtual Environments

**Figure 4:**
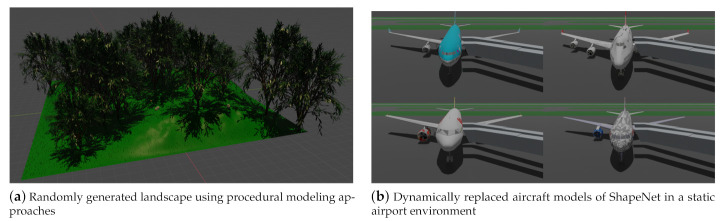
In (**a**) a procedurally generated landscape with vegetation is shown. A semi-static scene varied by aircraft models from free repositories is shown in (**b**).

Blender already offers a rich tool chain for modeling, texturing, simulation of physical processes and animation of 3D worlds in an appropriate way to define virtual environments for applications of LiDAR and Sonar measurements. The use of materials to define the reflectivity of a surface must be considered. Details on this are explained in Section 3.3.2. In the standard case, objects are modeled via meshes, parametric surfaces or Constructive Solid Geometry (CSG) in Blender and conceptualized to a scene. The standard Blender workflow allows a high degree of adaptability, but requires a lot of 3D modeling expertise and time to integrate enough variations into the training examples for AI. BLAINDER therefore provides several functionalities for quick generation and variation of virtual worlds by non-experts:

- For variation of a specific scene, the worlds can be generated completely or mostly procedurally. This is particularly useful for scene containing a lot of nature such as landscapes or vegetation (see Figure 4a).
- The worlds can be semi-static, i.e., an essential part is static even with advancing time (e.g., buildings, illustrated in Figure 4b using the example of an airport), while only a smaller part of the scene varies (e.g., specific aircraft models).
- By using animations, variation can be created within a topologically constant scene (translation of the sensor, movement of figures, physical simulation). See Section 3.1.2 for this.

For *procedural* variations, a separate script is provided for the automated generation of landscapes and their vegetation. Figure 4 illustrates such a landscape. Thus, the simulation can be executed in different natural environments. The script uses integrated Blender add-ons (The landscape is modeled using the extension ANT Landscape. For the insertion of trees, the add-on Sapling Tree Gen is used. The third add-on Add Mesh Extra Objects is used to generate rocks and stones) to create random landscapes with grass, trees, rocks and stones. Grass is inserted using Blender’s built-in particle system simulation tool. For this purpose, a 3D model of a blade of grass is loaded and placed at the positions specified by the particle system. This is necessary so that the sensor simulation recognizes the blades of grass as geometric objects. Please note that not every tree is regenerated. Instead, a tree may be generated only once where the tree geometry is shared between multiple tree instances for reasons of memory-efficiency.

#### 3.1.1. Semi-Static Scenes

In *semi-static* scenes, scene variation is achieved by replacing a smaller number of objects for each scene instance. Free repositories offer a wide choice of 3D objects for this case (see Section 1.1). The need for synthetic data in such applications has been demonstrated e.g., in [64], where also various sensors are simulated in a virtual environment, although no general framework for the creation of such 3D worlds is provided. Our add-on BLAINDER provides a principled methodology and open-source implementation for such use cases.

**Figure 5:**
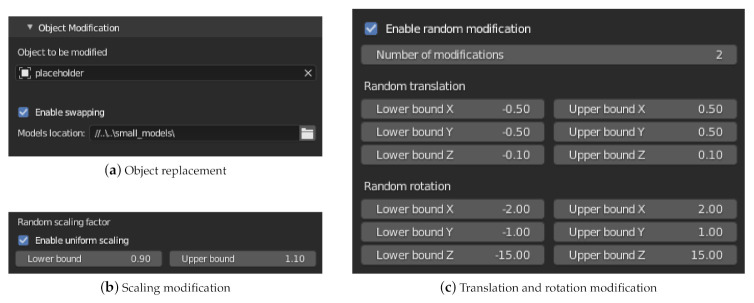
The shown menu allows the automated adjustment of a scene. The object to be modified can be exchanged (swap) with predefined models. In addition, the random modification of the property translation, rotation and scaling is possible.

The menu shown in Figure 5 allows the exchange and modification of objects. In the first part of the menu (Figure 5a), the user can select an object which is to be replaced by other models in scene variations. Supported file formats are *.fbx*, *.obj* and *.glb* or *.gltf*. Please note that each model must have an associated surface material that defines the reflectivity coefficient of the surface. Properties such as the position in the scene, animation paths or the semantic categorization remain unchanged for an alternative model, unless specified otherwise. Only the geometry of the object, the rotation and scale are set according to the definitions in the imported model.

In the second part of the menu (Figure 5b,c) the properties translation, rotation and scaling can then be modified. This modification can be used independently of the exchange of models from the first part of the menu. For each property and coordinate axis there is the possibility to set an interval for a random deviation. The translation and rotation properties are given as absolute values of the unit defined in Blender (tab Scene Properties). The value 0 means no change. Two types of modification are provided for scaling. With uniform scaling (Figure 5c), the scaling values in X, Y and Z direction are multiplied by the same factor. Otherwise, a separate, random factor is determined for each coordinate axis. The value 1 does not correspond to any change. Furthermore, the number of modifications per object can be defined. For each modification, a simulation run will be executed.

#### 3.1.2. Animations and Physics Simulation

Blender offers a variety of tools for creating and adapting animations (all related information about animation and physics simulation can be found in the Blender documentation). By using these tools, dynamic scenes can also be simulated using the add-on. One way of configuration is the use of key frames. With these key frames, the properties of objects can be defined for each point in time or calculation step. Between the time steps the interpolation of the values is done according to the selected interpolation method. Furthermore, constraints are also suitable for various animation tasks. A path object is specified in the condition of the object to be animated to describe the path of the movement. These two methods can be used to move all objects in a scene. This also includes the sensor. This allows camera movements, for example to simulate a LiDAR sensor on a vehicle.

Blender also features an elaborate physics simulation. This includes, among other things, the movement of objects under the influence of force, substance and fluid simulations as well as collision handling. Scenes that contain such physics simulations are also compatible with the BLAINDER add-on.

### 3.2. Virtual Sensors

#### 3.2.1. Predefined Sensors

**Figure 6:**
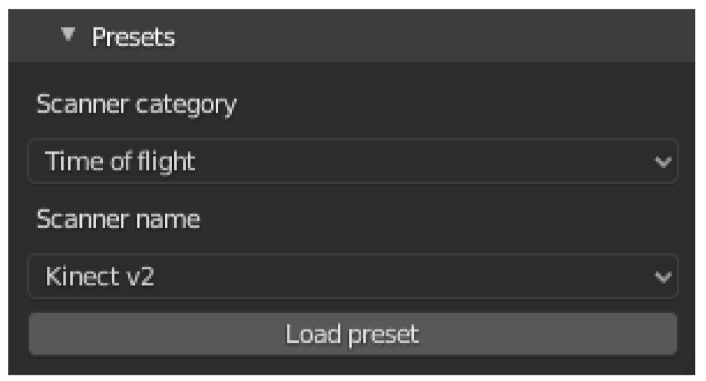
Screenshot of the Sensor Presets Selection Menu. After selecting a sensor category, the sensor name can be used to select a preset. By pressing the button to load the preset, the preset is accepted.

To simplify the use of the add-on, presets are provided for several common sensor models. Figure 6 shows a screenshot of the selection menu. The upper part of the menu shows one of the three categories LiDAR, Sonar and ToF. The respective sensor can be selected from the second part of the menu. After pressing the button to load the preset, the stored values will be applied. Further sensors can be added in a configuration file.

All presets are stored within a YAML-file (https://github.com/ln-12/blainder-range-scanner/blob/main/range_scanner/ui/presets.yaml (accessed on 17 March 2021)). This file contains the sensors listed in Table 1 with information about distance, resolution, field of view (FOV) and more. Own sensors can easily be added to this file, to integrate them in simulation.

#### 3.2.2. Adding Noise

As described in Section 2.3.5, random noise can be applied to the virtual sensor measurements, reflecting, e.g., tolerances of the measuring device or the influence of weather. In the current implementation, Gaussian noise according to the normal distribution can be added to the virtual sensors. The corresponding function of the Python library NumPy [65] is used for this purpose.

**Figure 7:**
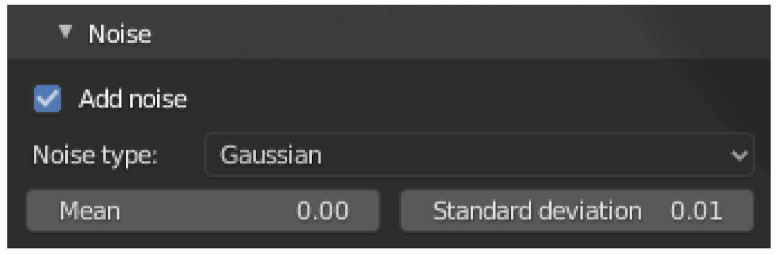
The screenshot shows the menu for configuring the random error. Here the normal distribution can be selected and parameterized by the mean and standard deviation.

Figure 7 shows the user interface section for configuring the random noise.

### 3.3. Signal Processing and Physical Effects

Section 2 discussed the basic principles of light and sound propagation on which the BLAINDER add-on is based. In the following, some implementation details are explained. A global, Cartesian coordinate system is assumed. Starting point for ray tracing is the determination of the origin and the direction of a ray. Both parameters are determined in the scene by a camera object selected by the user, which works as the active sensor. The origin of the rays equals the position of the sensor object. The direction of the rays depends on several factors. For each ray, the rotation of the sensor is set according to the sensor configuration. Each sensor type (see Section 2) has a field of view which is defined by an aperture angle $\alpha_{h}$ in horizontal and $\alpha_{v}$ in vertical direction. The azimuth angle then runs in the interval $\left[-\frac{\alpha_{h}}{2},+\frac{\alpha_{h}}{2}\right]$. The interval $\left[-\frac{\alpha_{v}}{2},+\frac{\alpha_{v}}{2}\right]$ applies accordingly to the polar angle. The step size between two measurements is the resolution of the sensor in the respective direction.

**Figure 8:**
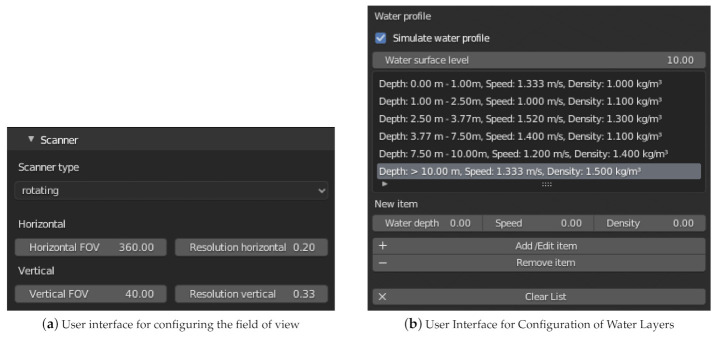
(**a**) The screenshot shows a menu for configuring the scanner type. (**b**) The figure shows a screenshot of the configuration menu for defining water layers.

Figure 8a shows a menu for configuring the scanner type. Furthermore, the FOV and the resolution can be set. The type can be one of the options rotating, static, or side-scan [46]. Depending on the sensor, different configuration options are available for the sensor’s field of view. The first two sensors have a field of view in horizontal and vertical direction, while the side-scan sensor only has a downward opening angle.

BLAINDER’s functionalities for emitting and following rays are implemented with the help of Blender’s ray casting function and helper functions for the automatic generation of a bounding volume hierarchy (The class BVHTree provided by Blender is used in the implementation). The raycast function’s parameters are the origin and direction of the beam as well as a maximum distance. The function returns a tuple with one vector each describing the intersection point and the surface normal at this point, the number of the polygon on which the intersection occurred, and the distance between the beam origin and the intersection point. To determine the first object hit, ray casting is performed for each object and the object with the shortest distance is selected.

Then the object surface at the position of the intersection is evaluated. The parameters of the material are used for this. If the material is diffuse, the intensity of the reflected beam is calculated using the reflectivity. In the case of a specular or transparent surface, additional rays are emitted from the original intersection point to simulate reflection or transmission. If these rays hit other objects, the surface material is evaluated again. Thus, the recursive calculation method introduced in Section 2 is applied. To simulate the measurement errors described in Section 2, the distance covered by the beam is stored in each recursion step. When the recursion is finished, all distances are summed up. This sum is then used to determine the depth value in the original direction of the beam. For the simulation of a two-dimensional side-scan Sonar, the three-dimensional data points are projected into the plane at the end. After each measurement, the direction of the beam is adjusted horizontally and/or vertically according to the sensor configuration.

#### 3.3.1. Sound Profile in Water

In Section 2.2.1 several factors were mentioned, on which the speed of sound and thus the propagation of a sound wave depends when traveling through water. The different velocities and refraction coefficients of the water layers may lead to continuous refractions during propagation. This continuous change is approximated by discrete steps. One possibility of modeling this in Blender is the use of cuboids in combination with glass material. To simplify the specification, a separate menu is provided in the user interface, as shown in Figure 8b. The figure shows a screenshot of the configuration menu for defining water layers. The upper part shows already entered data pairs. Below that the data sets can be added, edited and deleted. The water depth refers to the Z-value of the water surface level. For each data pair the speed and density of the water surface level must be specified.

For each water layer, the velocity and density of the water in this layer can be specified in this menu. The propagation of sound within water is simulated piecewise for each layer. In analogy to optical ray tracing, sounds are modeled as rays. If the sound ray hits an object within a layer, then the sound is reflected back, and no further water layers are considered. If the sound does not hit an object, it is refracted at the next boundary layer according to the water layers defined by the user. Equations (11) and (13) are used to determine the angle of refraction and the transmitted energy.

#### 3.3.2. Modeling Surface Properties with Materials

The software Blender contains comprehensive tools to describe the surface properties of objects. As common in 3D computer graphics, so-called ”materials” are used for this purpose. In addition, networks of so-called shaders can be created in Blender. A shader converts input parameters into surface properties. Each node of this network (shader nodes) fulfills a specific task. The output of a node (right side) can be used as input of another node (left side).

For the modification of object surfaces using shaders and the creation of the described networks, expert knowledge is required. In addition, the material properties do not necessarily indicate the reflectivity of a surface. To make the use for inexperienced users possible, this procedure is simplified. Regarding the simulation, the various physical parameters can be summarized using a single reflectivity factor.

This factor can be configured via the base color. The animation system integrated in Blender is available to change the value over time. The desired color values can be specified at key frame time.

The surface of an object is not always homogeneous. Water, for example, can influence the reflectivity at some parts of a surface. For this, textures can be applied instead of a homogeneous base color.

By setting the appropriate alpha value on the texture, different reflectivity values can be assigned to specific locations on the surface.

Transparent media such as glass or water not only reflect but also refract light rays. For a better distinction for the user, the glass shader can be used to define such a material. The refractive index *n* is defined by the value *IOR*. This allows the modeling of different objects such as glass panes or puddles. The latter represent a difficulty in the evaluation of LiDAR point clouds, because objects may appear below the ground due to reflections [66].

### 3.4. Semantic Labeling: Object Category

**Figure 9:**
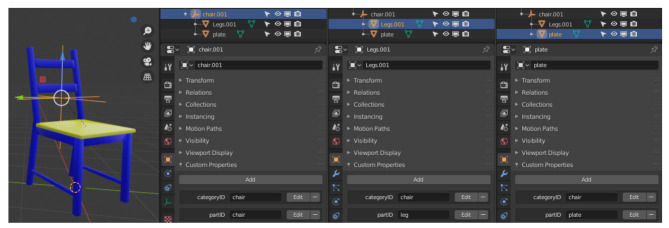
The screenshots show the approach to mark different objects and their components for classification by the add-on. All parts of the same object or object group (here: chair ) get the same *categoryID*. To distinguish the parts, the attribute *partID* is used.

The main purpose of the add-on developed in this work is the generation of point clouds with semantic labels for different objects and object components as training data for ML algorithms. This requires configuration by the user and can be done in two ways. Each object can be assigned the two properties *categoryID* (name of the object) and *partID* (name of the object component). Figure 9 illustrates this using a chair as an example. The left figure shows the chair. The classification of the whole object is shown next to it. In the second image from the right, the chair legs are classified, in the right image the seat plate. It can be seen that the chair as a whole, the seat and the chair legs have been named individually.

The chair object consists of an empty placeholder object. It has the type plain axes and groups the child geometries using parent-child relationships. Both the chair legs and the seat are created as separate objects. Each of the three objects has different attribute values to express the association with the respective group.

**Figure 10:**
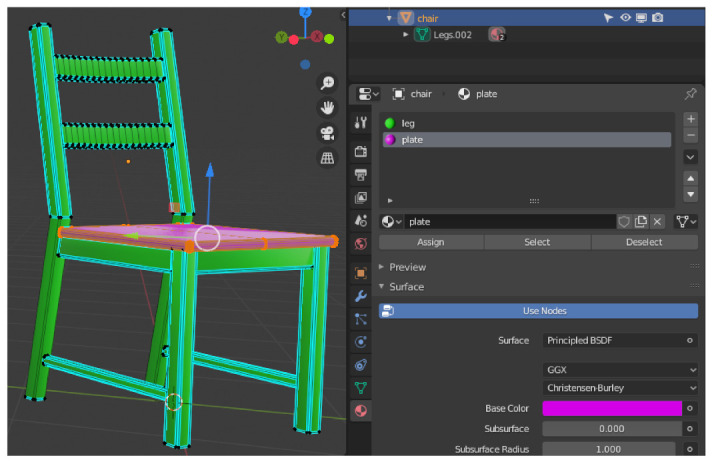
On the left half of the image you can see a chair object in edit mode. All points and lines belonging to the chair surface were selected and classified using the button for assigning a material. The chair legs were also assigned to a material. The group affiliation is defined by the respective material name.

An alternative way of classification is the assignment of materials shown in Figure 10. This can be used when the object is a coherent geometry and no separation into sub-objects is desired. In the edit mode of Blender, all vertices belonging to an object group are selected. Afterwards the material is assigned by pressing the corresponding button. The assignment to an object group is determined by the assigned material name.

The attribute *categoryID* must be specified for each object. If this attribute is missing, the object name is used as a substitute. If the attribute *partID* is set, it is used for the classification. Otherwise, the material is used to determine the class. In the output formats, the different classes are represented by different numerical values.

### 3.5. Data Export

After the simulation of a virtual sensor, the generated point-cloud is available as internal data structure of the add-on. For use outside of Blender, the export as a savable file is therefore necessary. A point-cloud consists of data records for all points, each containing the following information:

- point position in space (X-, Y- and Z-coordinate)
- semantic label
- intensity of the measuring point
- color of the object surface
- distance between sensor and object surface

There are different data formats for storing such measurement data. For example, the LAS format (The current version 1.4 of the specification was released in November 2011 (December 2020)) was developed by the American Society for Photogrammetry and Remote Sensing for handling three-dimensional LiDAR point clouds [67]. It contains 11 point data record formats to cover different requirements. Details can be found in the specification [68]. For the implementation in the context of this paper record format number 2 is used. To simplify the handling of the binary LAS data format, the Python library laspy [69] is used.

The generated data will be used in machine-learning applications. In this area the binary hierarchical data format (HDF) is often used. This format was designed by the HDF Group and is characterized by the support of heterogeneous data, the self-describing structure, the possibility to store metadata and the support of a variety of software packages [70]. The HDF5 data format supports two basic object types. Datasets contain the raw data and metadata. Groups are used to organize the datasets and subordinate groups. Together, these two types of objects enable a structured, hierarchical structure of the data [71]. For the integration into Python the library h5py [72] is used.

In addition to these two binary formats, the possibility of saving in text form is also implemented. For this purpose, the data can be stored in the CSV format. In this format, each row contains one data record. The header row contains the column names.

The different data formats can be selected independently. Additionally, an option is available to specify the way animations are saved. These can be saved in two ways:

- **Single frames:** For each animation step a separate point-cloud is created. Since the LAS and CSV formats do not allow a hierarchical structure, one file per time step is generated. In contrast, the representation of a time step in HDF format is done by one line in the data set. Please note that the length of the lines may vary. Therefore, a variable length array must be used as data type.
- **Summarized:** All animation steps are also simulated separately. The difference, however, is that all data is summarized in a point-cloud at the end and stored in a single file.

For depth cameras there are visualization options in addition to the previously mentioned export options for raw data. These include a rendered image of the scene, a segmented image including the description of all image elements and a depth image.

Besides point clouds and depth images, annotated RGB images can be created for visualization of the scenes seen by a depth sensor. Blender’s camera object is used for creation of such color images. Further settings can be made in the tab Render Properties of Blender. In addition to computer graphics renderings, semantically segmented images can be created where pixel colors represent object classifications. Also, an image description can be generated in text form for each image. For this purpose, the library PASCAL VOC Writer [73] is used. The program ImageSet Viewer [74] is suitable for viewing the annotations. As a third visualization of the point-cloud a depth image can be generated. This requires the configuration of the minimum and maximum depth values. All distances below the minimum are displayed in white, above the maximum in black. For distance values in between, the color value is interpolated linearly.

## 4. Results

### 4.1. Depth Cameras and Range Scanners (LiDAR, ToF)

**Figure 11:**
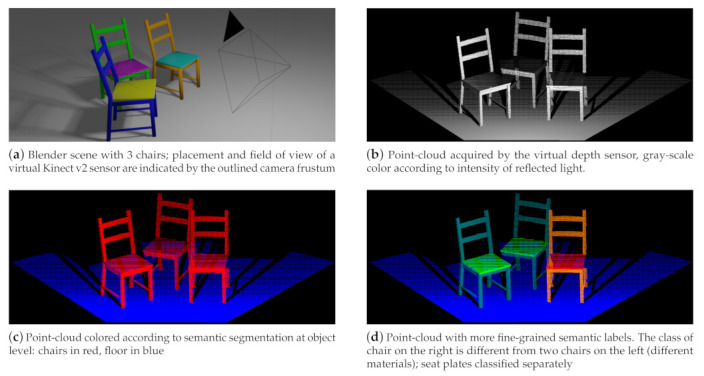
A simple scene in Blender (**a**) and three visualizations of a point-cloud generated by a virtual depth sensor. The bottom row shows two alternative semantic labelings.

The core purposes of the add-on BLAINDER is the automatic semantic segmentation of objects and object components in point clouds generated from virtual depth sensors. Figure 11 shows a simple scene in Blender that contains three chairs (Chair model from [75]). On the top left, also the placement and field of view of a depth sensor is shown. The bottom row shows two alternative semantic labelings of a scene. BLAINDER’s default settings for the Kinect v2 depth sensor were used in this example.

In Figure 11b–d, the three point-cloud visualizations show black stripes behind the chairs along the floor. These correspond to the shadow areas of the light beams emitted by the measuring device. All other black areas do not contain any data because they are outside of the field of view of the measuring device. Figure 11b shows the point-cloud acquired from the virtual sensor where the gray-scale color of points indicates the intensity of the light reflected back to the sensor. Here, points closer to the sensor are shown lighter, while farther way points are darker.

Figure 11c illustrates the assignment of the points to object categories. Points assigned to the same categories get the same color. This visualizes all chairs in red and the floor in blue.

In accordance with the procedures described in Section 3.4, a more fine-grained categorization is also possible. For Figure 11d, the seat and frame of the left and middle chairs were each given identical categories. The parts of the right chair were assigned different categories. The user can choose whether corresponding parts of different objects should be treated as one group (left and middle chair) or as different groups (middle and right chair).

### 4.2. Semantically Labeled 2D Images

**Figure 12:**
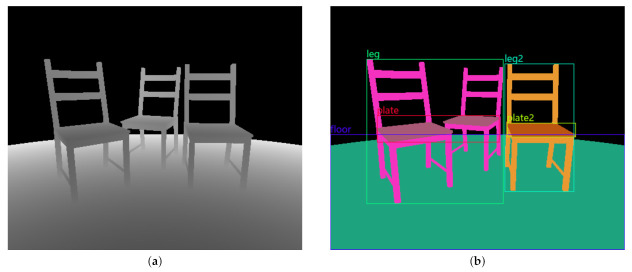
(**a**) Depth image of the example scene where pixel colors correspond to distance measurements. (**b**) Pixel-wise semantic segmentation of the image. The left and middle chair components share the same semantic category and are therefore colored identically.

In addition to three-dimensional visualizations of the simulated measurement data as point clouds, two-dimensional images can also be generated using the add-on for ToF sensors. In the depth image shown in Figure 12a, gray-scale pixel colors represent the distance to the object seen by the virtual sensor. Here, the lighter the color of an image pixel, the further away the corresponding location of the object is from the camera. The black area in the background indicates the lack of data due to the spatially limited measuring range.

Pixel-wise semantic segmentations of images may be augmented with object bounding boxes and textual descriptions of the object category, similar to the PASCAL VOC [39] data set. An example is shown in Figure 12b (To display the image description in Figure 12b the program ImageSet Viewer [74] was used). Here, the same fine-grained semantic labeling as in Figure 11d is shown.

### 4.3. Animations

For the creation of animations, the tools available in Blender can be used. Animations are managed by Blender and taken into account during the simulation of virtual sensors. As explained in Section 3.1.2, both the animation of scene objects and the virtual sensors is supported by BLAINDER.

**Figure 13:**
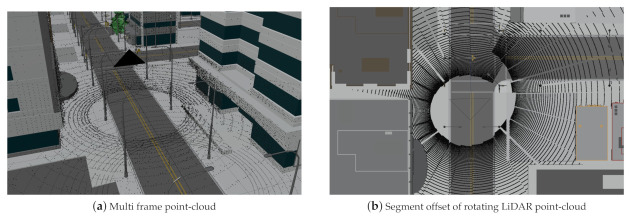
(**a**) A simulated 360°-LiDAR sensor travels along a street of a 3D city model (City model from to [76]), generating multiple point-cloud frames. (**b**) The top view shows the spiral shape of a point-cloud for a single frame which is characteristic for moving, rotating LiDAR sensors.

Figure 13 shows an example of a 360${}^{\circ }$-LiDAR sensor that follows a predefined motion path. For illustration purposes, the simulation is performed with 24 single steps per second and the sensor configuration of one complete revolution per second. This allows the characteristic spiral pattern to be seen.

### 4.4. Sound Navigation and Ranging (Sonar)

**Figure 14:**
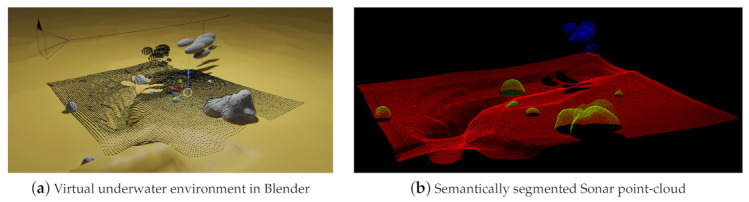
(**a**) The illustration shows an underwater scene modeled in the software Blender. (**b**) Point-cloud acquired from virtual Sonar sensor with semantic labels.

In addition to the simulation of depth cameras and range scanners, BLAINDER also supports the simulation of sound-based distance measurements (Sound Navigation and Ranging (Sonar)). The underwater scene shown in Figure 14a is used as an example for a side-scan Sonar measurement. The lake bottom is shown in yellow and has several gray boulders laying on it. Additionally, gray ellipsoids above the bottom serve as a schematic swarm of fish. This fish swarm moves from left to right. The camera symbol in the upper left corner represents a side-scan Sonar sensor that moves along the black line from right to left through the scene. The black dots represented an accumulated point-cloud with measurements from all frames of the animation.

Figure 14b shows the resulting semantically segmented point-cloud where fish are shown in blue, boulders in green, and the lake bottom in red.

In Blender, a lake bottom was modeled with a yellow color and several gray boulders were placed on it. Also, a group of gray ellipsoids was added to the scene to represent a swarm of fish. This group moves from left to right during the measurement. A virtual side-scan Sonar sensor was also added to the scene that moves from right to left along the curved black line during the simulation. Thus, the fish swarm and the sensor move in opposite directions during the simulation. The virtual side-scan Sonar generates depth measurements along a 2D scan line in each frame. The black dots in Figure 14a represent the Sonar measurements accumulated over the complete animation. It can be seen that the accumulation of points to the left of the fish swarm is smaller than the swarm itself. This can be explained by the opposite movement of the side-scan Sonar and the fish swarm.

**Figure 15:**
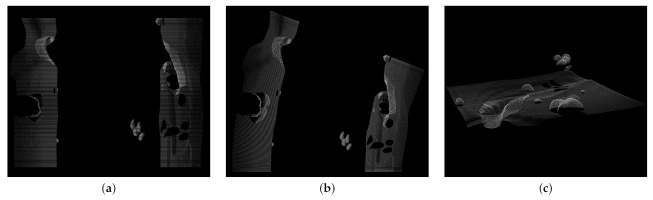
Display and export options for distance-measurement data of virtual Sonar sensors. (**a**) The left image corresponds to the classic side view Sonar. (**b**) For the middle image, the rotation of the measuring probe was taken into account for the alignment of the measurement data. (**c**) The right image shows the intensity measurement data as a 3D point-cloud.

In addition to semantically labeled point clouds, BLAINDER is also able to generate different kinds of 2D depth images and point clouds with distance-measurement information, as commonly provided by real Sonar sensors. The three available output modes are shown in Figure 15. All images show the intensity of each measuring point by using differences in brightness. Again, bright pixels represent a strong echo signal, dark pixels represent a weak signal and black pixels represent unrecorded measurement data. Figure 15a shows the measurement data without spatial alignment to the sensor’s movement. Here, each line in the image corresponds to a 2D scan line taken by the sensor at one time step. The second display mode in Figure 15b also shows the intensity data as 2D depth image but here the sensor orientation is accounted for. It can be seen that the measuring probe has moved along a curve. Figure 15c shows the third option for data visualization and export. In this case, the data is provided as a point-cloud with intensity information for each point.

## 5. Evaluation

### 5.1. Validation of Measurements

For assessment of the quality of the virtual sensors’ measurements two illustrative experiments are presented in the following. The first experiment compares a depth map acquired from a real-world scene with a depth map obtained in a virtual environment modeled to match the real environment. The second experiment compares two point clouds of a large environment where one point-cloud was acquired by using a high-detail scan of a real-world mine and the other point-cloud by virtually scanning a polygonal 3D model of the mine.

**Figure 16:**
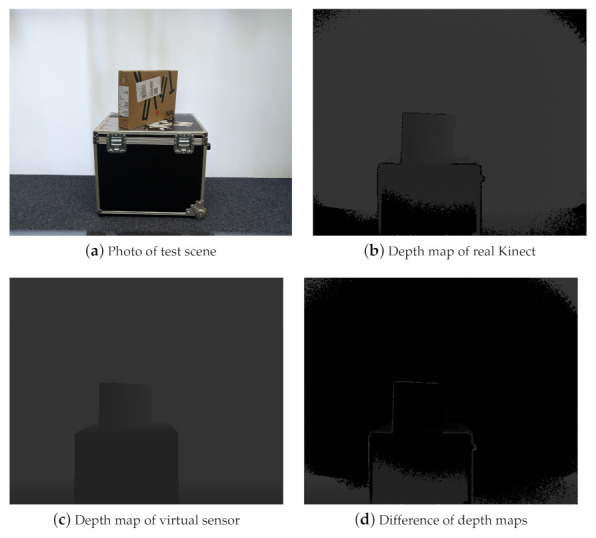
(**a**) Simple real-world test scene with two stacked boxes. (**b**) Depth map acquired by the Kinect v2. Vignetting effects at the periphery and edge noise can be observed as black pixels. (**c**) Depth map acquired by a virtual sensor with a simple 3D model of the test scene. (**d**) Difference image of the two depth maps.

The first experiment involves a simple test scene consisting of two boxes stacked one on top of the other. Figure 16a shows the real-world set-up. Figure 16b shows the depth map of this scene obtained by a Kinect v2 sensor. The lighter a pixel, the larger is the distance to the sensor. If a pixel is totally black, no depth value could be acquired by the sensor (Such invalid measurements will not contribute to the point-cloud extracted from the depth map). In the depth map, such black pixels can be observed at the periphery due to vignetting and at hard edges (“edge noise”) in the image which have been reported as typical forms of noise for the Kinect v2 sensor [77]. Figure 16c shows the depth map of a virtual environment that was manually modeled in Blender. The 3D model does not contain all geometric detail of the real-world scene. Also, it does not contain textures which could be used to capture the varying reflection properties on the boxes’ surfaces, e.g., due to stickers. A difference image of the two depth maps is shown in Figure 16d. Differences occur mainly in areas where the physical sensor suffers from vignetting and edge noise and, to a smaller extent, at the partly (specularly) reflective top of the box, due to simplified modeling as perfectly diffuse surface in the 3D model. At all other places, differences between the real and virtual sensor are not noticeable. Clearly visible are the differences at the periphery and at the objects’ silhouettes where the physical Kinect sensor suffers from noise. Similarly, regions where the 3D models possess less geometric detail, e.g., the knob on the right of the lower box, are well visible. Smaller differences can be noticed at the top surface of the large box whose varying reflective properties are not accurately captured in the simple 3D model. In all other regions, the correspondences between the two depth maps are very high.

**Figure 17:**
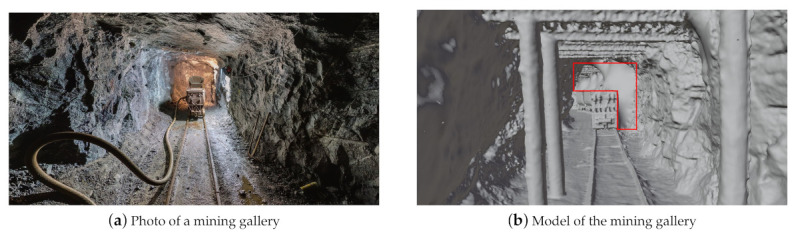
(**a**) The photo shows a gallery of the mine Reiche Zeiche in Freiberg. Graphic after [78]. (**b**) The photo shows a polygonal 3D model of the gallery Wilhelm-Stehender-Süd in the mine Reiche Zeche in Freiberg in which a virtual depth sensor was simulated. The red area marks a region where additional polygons were added to close holes present in the initial photogrammetric model.

The second experiment involves a large environment where a moving depth sensor was simulated. For this, data from the research and training mine *Reiche Zeche* in Freiberg was used. A reference point-cloud of a mining gallery was provided by the Institute for Mine Surveying and Geodesy of the TU Bergakademie Freiberg that was acquired using a Riegl LiDAR scanner in the underground mine. Furthermore, a polygonal 3D model of the mine was provided by the Institute of Computer Science of the TU Bergakademie Freiberg. The polygonal model was generated using a photogrammetry method from color and depth images of a Kinect sensor. In some areas that were not captured by the scanner, the initial photogrammetric model contained holes that were filled in a post-processing step. A section of the polygonal model is shown in Figure 17b where the area marked in red is an example of a spot where holes were filled in a manual 3D modeling process. Likewise, surfaces were inserted at both ends of the tunnel to obtain a closed model. In such areas, deviations from the reference point-cloud must be expected.

The simulation took place in the photogrammetric, post-processed 3D model. A total distance of about 50 m was scanned using a virtual depth sensor. The resulting point-cloud was then compared with the reference data in the CloudCompare software. To do this, the two point clouds were first approximately aligned by hand. Then, the registration was refined within CloudCompare by an Iterative Closest Point Algorithm (ICP). The two aligned point clouds were then compared using the CloudCompare tool “Cloud-to-Cloud Distance”. The reference point-cloud contains 7.2 million points, the simulated point-cloud 1.9 million points.

**Figure 18:**
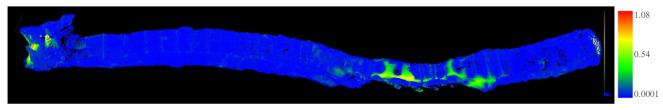
The image was created using the CloudCompare software. A comparison of the reference point-cloud with the simulated point-cloud was performed based on the integrated tool Cloud-to-Cloud Distance. At the right edge of the image a scale with the absolute deviation in meters is shown. This ranges from 0.00 (blue) to 1.08 m (red).

Figure 18 visualizes the distances between the two point clouds where the scale on the right side shows absolute differences in meters. Most of the simulated points have a distance of less than 0.067 m to the reference point-cloud. Larger deviations occur as expected between the train and the tunnel ceiling and at the ends of the tunnel, because extra polygons were added here during hole-filling. Regarding this comparison, it must be considered that both models have some errors from the outset. The overall excellent fit indicates, however, that both approximate the ground-truth geometry rather well. For the purpose of evaluating BLAINDER’s virtual sensor simulations, we conclude that BLAINDER both provides accurate sensing capabilities for single shots and can correctly integrate a series single-scans by a moving sensor into an integrated point-cloud.

### 5.2. Runtime Performance

Although the generation of semantically labeled point-cloud data is usually not real-time critical, too large runtimes are clearly not desirable in practice considering the need for ML applications for large amounts of training data. This section therefore provides some insights into the runtime behavior of BLAINDER’s virtual depths simulations. All experiments were carried out on a desktop PC with processor: Intel Xeon E3-1231 v3 @ 3.40 GHz, working memory: 16.0 GB DDR3 @ 1600 MHz, graphics card: Radeon RX 590. Generally, the duration of a depth sensor simulation depends on the number of rays emitted and the computation time for each ray. The latter may vary however strongly depending on the complexity of the particular scene. To exclude such influences, some of the experiments use simple synthetic test scenes, whose complexity can be increased in a controlled fashion, where rays only interact with one surface each. Options for animations, weather influences, visualizations or file exports were disabled, unless otherwise stated.

#### 5.2.1. Number of Measurement Points

Available depth sensors vary widely in their spatial resolution. At the high-end, e.g., the Azure Kinect ToF sensor offers a 1-Megapixel resolution, i.e., approximately $10^{6}$ depth measurements. In BLAINDER, depth measurements are obtained by casting rays into the scene, one ray per data point. Thus, an increase of the computation time that is linear to the number of data points can be expected. The first experiment was designed to test whether a linear runtime increase can be maintained during the simulation of sensors with a spatial resolution similar to today’s high-end sensors.

**Figure 19:**
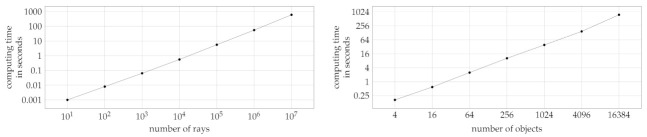
The diagrams show the computation time for a scene consisting of a camera and a square area as a function of the number of simulated rays (**left**) and, resp., number of objects (**right**).

For this test, a sensor with a horizontal and vertical field of view of 90° each was used. The sensor’s spatial resolution was varied during the experiment. Orthogonal to the direction of view of the sensor, a planar, square surface consisting of four vertices was placed in the scene that covers the entire field of view of the sensor. As the simulation includes certain constant start-up costs, an initial simulation was performed with one ray only. For this, a reference value of 0.019 s was obtained. For time measurements with multiple rays, this reference value was subtracted as start-up costs for the first ray. Diagram Figure 19 (left) shows the results. It can be seen that there is a linear relationship between the number of simulated rays and the computation time. A tenfold increase in the number of rays results in a tenfold increase of the duration.

#### 5.2.2. Number of Objects

Besides the linear increase with the number of rays, the computation time is also expected to increase (at least) linearly with the number of objects in the scene. This is because each ray must be tested at least once against each object (or its bounding volume). For geometrically complex objects, e.g., trees with many leaves, each ray must potentially be tested against many sub-objects and ultimately polygons. In order exclude aspects of geometric detail, again a simple test scene was designed to determine the influence of the number of objects on the runtime performance.

Again, a depth sensor with a square field of view of 90° each was used. The spatial resolution was fixed at 300 × 300 rays. A series of scenes with an increasing number of objects was then defined as follows: The first scene contains just one square surface orthogonal to and fully covering the sensor’s field of view. Then the square object was repeatedly subdivided into 4 smaller square objects. This yields a series of scenes where the number of objects is $4^{i}$ with i∈{0,1,2,3,4,5,6,7}. This design ensures that each ray intersects exactly one object. Similar to the number of rays the diagram Figure 19 (right) shows a linear increase. If the number of objects is quadrupled, the calculation time increases by this factor.

#### 5.2.3. Weather Simulation

To measure the influence of the weather simulation on the computation time, the city scene shown in Section 4.3 is used together with the sensor configuration “Velodyne UltraPuck”. The computation without weather influence took an average of 17.05 s. The computation with the simulation of dust was only slightly slower and took 18.59 s. The computation takes longer when simulating rain with an average of 44.33 s. The variation of the specific weather parameters has no effect on the runtime behavior.

#### 5.2.4. Comparison to Similar Applications

**Figure 20:**
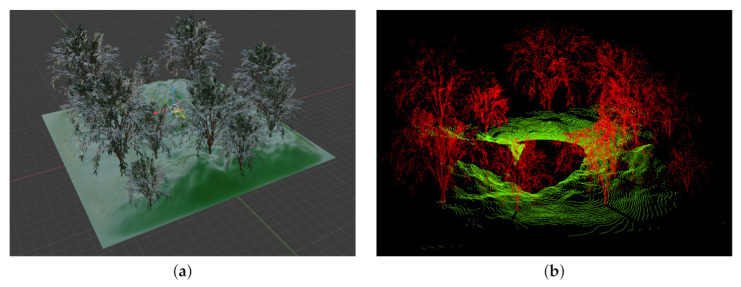
(**a**) Landscape scene with multiple trees. The simulated UltraPuck sensor is positioned in the center of scene. It has a horizontal field of view of 360° and vertical field of view of 40°. (**b**) Semantically labeled point-cloud generated by BLAINDER. Trees in red, ground in green.

The Helios simulation tool (see Section 1.1) is used for a speed comparison. The comparison is based on a landscape scene shown on the left of Figure 20. The simulated UltraPuck sensor has a horizontal field of view of 360° with a resolution of 0.2° and a vertical field of view of 40° with a resolution of 0.33°. For testing Helios, the source code of the GitHub entry 53f074b was used in combination with the single ray configuration (see [79]).

For converting the scene from Blender to a format readable by Helios, the extension Blender2Helios [38] was used. The configuration file created by this utility has been adjusted according to the sensor characteristics to create equal initial conditions. All measurements were performed without the Helios user interface (headless mode). Figure 20, right, shows the generated point-cloud of the BLAINDER simulation. The point-cloud generated Helios is essentially identical. Although Helios needs on average 8.43 s, the computation by the extension described in this paper takes on average 4.64 s.

## 6. Conclusions and Outlook

With the development of BLAINDER we have succeeded in extending the open-source 3D modeling software Blender by an add-on for LiDAR and Sonar simulations. The add-on provides both physically based depth sensor simulations and functionalities for semantic augmentation of 3D worlds. It can be used to generate a broad base of synthetic reference data for AI-supported evaluation of point clouds and images for applications where the database is not large enough for training.

The current status of development offers starting points for future extensions and optimizations. These include, on the one hand, more forms of scene variation to extend procedural and semi-static approaches, and, on the other hand, more granular error models to represent sensor behavior. External factors were described, which can influence distance measurement. Here the implementation of more complex computational models and extension by factors not considered so far is conceivable. Incorporating an advanced error model for ground fog as a meteorological influence for LiDAR is part of our current research work. Furthermore, the propagation of sound waves is currently approximated by rays in the Sonar simulation. The physically correct simulation of a wave could improve the result. Radar measuring devices are also frequently used in the automotive industry and help the user, for example, when parking. These were not considered in the context of this paper and can be implemented as an additional sensor class in the future.

The simulation quality was evaluated in Section 4. Both the calculation speed and the simulation accuracy are satisfactory. Section 5.2.4 shows that the simulation within the tested scene is faster with the developed extension than in a software with comparable functionality. Nevertheless, there is potential for optimization in the calculation speed. The software libraries CUDA, DirectX and Vulkan allow the outsourcing of ray tracing calculations to the GPU to execute them faster than on the main processor. For this purpose, however, it is necessary to investigate to what extent such a solution can be implemented without recompiling Blender.

## Figures and Tables

**Table 1:** List of predefined sensors in BLAINDER saved in the presets YAML-file.

Name	Category	Type
Generic LiDAR	LiDAR	rotating
Generic Sonar	Sonar	sideScan
Velodyne UltraPuck	LiDAR	rotating
Velodyne AlphaPuck	LiDAR	rotating
Microsoft Kinect v1 (default mode)	ToF	static
Microsoft Kinect v1 (near mode)	ToF	static
Microsoft Kinect v2 (default mode)	ToF	static

## Abbreviations

The following abbreviations are used in this manuscript:

AI	Artificial Intelligence
BRDF	bidirectional reflectance distribution function
BSDF	bidirectional scattering distribution function
BTDF	bidirectional transmittance distribution function
CSG	Constructive Solid Geometry
FOV	field of view
HDF	hierarchical data format
ICP	Iterative Closest Point Algorithm
LiDAR	Light Detection and Ranging
ML	Machine Learning
Radar	Radio Detection and Ranging
Sonar	Sound Navigation and Ranging
ToF	Time-of-Flight
